# Epstein–Barr virus DNA change level combined with tumor volume reduction ratio after inductive chemotherapy as a better prognostic predictor in locally advanced nasopharyngeal carcinoma

**DOI:** 10.1002/cam4.4964

**Published:** 2022-07-19

**Authors:** Zhong‐zheng Xiang, Tao He, Yuan‐yuan Zeng, Fang Liu, Bian‐fei Shao, Tian Yang, Jia‐chun Ma, Xi‐ran Wang, Si‐ting Yu, Lei Liu

**Affiliations:** ^1^ Department of Head and Neck Oncology Cancer Center, West China Hospital of Sichuan University Chengdu Sichuan P.R. China; ^2^ State Key Laboratory of Biotherapy West China Hospital of Sichuan University Chengdu Sichuan P.R. China; ^3^ Department of Radiation Oncology Cancer Center, West China Hospital of Sichuan University Chengdu Sichuan P.R. China

**Keywords:** Epstein–Barr virus DNA change level, inductive chemotherapy, nasopharyngeal carcinoma, prognosis, tumor volume reduction ratio

## Abstract

**Background:**

To explore the prognosis predicting ability of the combined factors, Epstein–Barr virus DNA change level (EBVCL) and tumor volume reduction ratio (TVRR) after inductive chemotherapy (IC), in locally advanced nasopharyngeal carcinoma (LANPC).

**Methods:**

From 2010 to 2018, 299 LANPC patients were included in this retrospective study. Receiver operating characteristic (ROC) curve analysis was performed to acquire the best critical values. According to the best critical values of EBVCL and TVRR, patients were stratified into low‐ and high‐risk groups. Kaplan–Meier and ROC curve analyses were utilized to verify the prognostic ability of the new predictor (EBVCL+TVRR). The prognostic values among EBVCL+TVRR, EBVCL, TVRR, TNM stage, and the RECIST 1.1 criteria were compared by ROC curve. The primary end points were overall survival (OS), progression‐free survival (PFS), distant metastasis‐free survival (DMFS), and locoregional failure‐free survival (LRFFS).

**Results:**

ROC curve analyses of TVRR on three‐year survival showed the best critical values of TVRR was 32.72% for OS, 30.21% for PFS and LRFFS, 29.87% for DMFS. The best critical value of EBVCL was 127 copies/ml for OS, and 87.7 copies/ml for PFS, DMFS, and LRFFS. The three‐year OS, PFS, DMFS, and LRFFS for low‐ and high‐risk groups were 97.7% versus 78.3% (hazard ratio [HR] = 0.2398; 95% confidence interval [CI]: 0.1277–0.4502; *p* < 0.0001), 91.1% versus 60.9% (HR = 0.3294; 95% CI: 0.2050–0.5292; *p* < 0.0001), 94.2% versus 68.7% (HR = 0.2413; 95% CI: 0.1284–0.4535; *p* < 0.0001) and 97.8% versus 77.9% (HR = 0.3078; 95% CI: 0.1700–0.5573; *p* = 0.0001), respectively. The maximal area under ROC curve of EBVCL+TVRR, EBVCL, TVRR, TNM stage, and RECIST 1.1 criteria for three‐year OS was 0.829, 0.750, 0.711, 0.555, and 0.605, respectively.

**Conclusion:**

The new‐developed indicator (EBVCL+TVRR) could better predict the LANPC patient's survival after IC compared with TNM stage system or RECIST 1.1 criteria.

## Graphical abstract

EBVCL+TVRR could effectively screen out the high‐risk population after inductive chemotherapy for LANPC. EBVCL+TVRR was identified as a better independent prognostic indicator for LANPC compared with TNM stage system or RECIST 1.1 criteria.

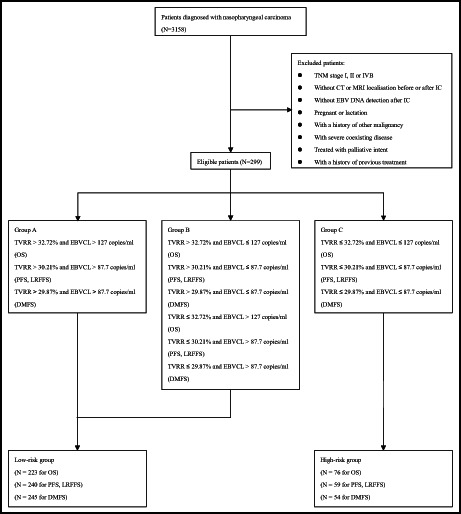

## 1 INTRODUCTION

Nasopharyngeal cancer (NPC) is a relatively uncommon malignant tumor worldwide but quite prevalent in South China, North Africa, and Southeastern Asia. In 2018, a total of 129,000 new NPC cases were reported by the International Agency for Research on Cancer.^1^ Seventy percent newly NPC patients were diagnosed as locally advanced stage.^2^ Inductive chemotherapy (IC) followed by concurrent chemoradiotherapy (CCRT) has been recommended to treat the locally advanced NPC (LANPC).^3^ For some previous studies conducted by Sun and Zhang et al. had illustrated that, when compared with CCRT alone, LANPC patients who received IC + CCRT possessed superior 3‐year failure‐free survival and overall survival (OS).^4^, ^5^

Currently, TNM (tumor‐node‐metastasis) stage system proposed by Union for International Cancer Control/American Joint Committee on Cancer (UICC/AJCC) is used to classify NPC and predict the patient's prognosis. But this anatomy‐based only TNM stage system is too inadequate to forecast the prognosis, hence, many studies have explored whether there exists other significant clinical factors combining with TNM system would better predict the survival. The plasma Epstein–Barr virus (EBV) is maybe the most frequently studied risk factor for NPC. For EBV DNA occurs in almost all undifferentiated or poorly differentiated NPC.^6^ Studies have also demonstrated that higher level of plasma EBV DNA is associated with poorer prognosis and early metastasis in NPC.^7^, ^8^, ^9^ And the pathogenesis of NPC is extremely correlated with EBV infection, circulating EBV DNA in plasma has been confirmed as an effective “liquid biopsy” method for NPC, which possesses a sensitivity of 96% and a specificity of 93%.^8^, ^10^ Lin‐Quan Tang et al. conducted a prognosis nomogram for NPC base on the pre‐treatment plasma EBV DNA, and found that this system owns superior predicting power than the system without combination of EBV DNA.^11^ Recently, Jiawei Lv et al. developed a system identifying prognostic phenotypes in NPC by tracking the circulating EBV DNA during sequential chemoradiotherapy, and revealed that the detection of EBV DNA level during treatment supplied more prognosis information.^12^

In addition, more and more attention has been paid to the tumor volume's prognosis ability in NPC, for which can more directly reflect the tumor's load. Studies have revealed that tumor volume could effectively predict the prognosis of NPC.^13^, ^14^, ^15^ Some other researchers also demonstrated that the tumor volume's prognosis predicting ability was even superior to the TNM stage.^16^, ^17^, ^18^ From the perspective of predicting the prognosis with the dynamic change of tumor volume, our previous studies have further proved that the tumor volume reduction ratio after IC is an effective indicator for patient's prognosis in LANPC.^19^

Herein, we explored the prognosis predicting ability of combined EBV DNA change level (EBVCL) with tumor volume reduction ratio (TVRR) after IC in LANPC for the first time, aiming to complement the TNM staging system with additional prognostic predicting information.

## 2 MATERIALS AND METHODS

### 2.1 Participants

This retrospective analysis enrolled patients who have received 2–3 cycles of IC combined with cisplatin‐based CCRT at West China Hospital of Sichuan University from July 2010 to June 2018. Before conducting this study, we acquired ethical approval from the Biomedical Ethics Committee of West China Hospital, Sichuan University, and the written informed consent was exempted (No. 2021–1341). Inclusion criteria were as follows: Age ≤70 year‐old, histopathologically diagnosed with LANPC, without distant metastasis, III‐IVA stage (8th edition stage‐classification of UICC/AJCC), Karnofsky performance scores ≥70, previously untreated, treated with curative intent, with both 3.0 mm scanning layer thickness enhanced computed tomography (CT) localisations or both magnetic resonance imaging (MRI) localisations within 2 weeks before and after IC, with detectable EBV DNA before IC, sufficient functional reserve of liver, renal, and bone marrow. Patients with the following situations were excluded: pregnant or lactation, previously suffered from malignancy, serious coexisting sickness, treated with palliative intent, or previously treated with chemotherapy, radiotherapy, or surgery.

Pretreatment evaluations were as follows: Complete case history, physical examination, laboratory examination, plasma EBV DNA level detection, nasopharyngoscopy, histologic biopsy, enhanced MRI and CT of the cervical and nasopharyngeal region, chest CT, abdominal CT and ultrasonography, and a body bone SPECT imaging scan. The 18F‐fluorodeoxyglucose‐positron‐emission tomography was conducted when necessary.

### 2.2 Chemotherapy

All patients completed 2–3 cycles of IC, the majority of regimens were TPF (docetaxel 60 mg/m^2^ on day 1, cisplatin 60–75 mg/m^2^ on day 1 or within 3 days, 5‐fluorouracil 600 mg/m^2^ per day on day 1–5) and GP (gemcitabine 1000 mg/m^2^ on day 1 and day 8, cisplatin 75 mg/m^2^ on day 1 or within 3 days), the others were TP (docetaxel 75 mg/m^2^ on day 1, cisplatin 75 mg/m^2^ on day 1 or within 3 days) and PF (cisplatin 80 mg/m^2^ on day 1 or within 3 days, 5‐fluorouracil 800 mg/m^2^ per day on day 1–5); all patients received 3 cycles of cisplatin‐based chemotherapy (cisplatin 75–100 mg/m^2^ on day 1 or within 3 days) during radiotherapy. Either IC or concurrent chemotherapy was administered every other 3 weeks.

### 2.3 Radiotherapy

All participants completed curative IMRT according to the reduced‐volume radiotherapy guidelines for NPC.^20^ The detailed target delineation of radiotherapy was described in our previously published paper.^19^ Briefly speaking, GTVnx represented the primary nasopharyngeal lesion, and GTVnd represented the positive cervical lymphonodus. CTV‐1 represents the clinical target volume in high‐risk region, which contained the GTVnx with a 5–10 mm margin and the entire nasopharynx. CTV‐2 represents the clinical target volume in low‐risk region, which included CTV‐1, the bony structures (such as skull base, pterygoid processesptery, and gopalatine fossa, etc), parapharyngeal space and retropharyngeal lymphatic drainage area. CTV‐N represents the clinical target volume of cervical lymphonodus regions (levels II, III, IV, V and Ib when necessary). The prescribed doses were the following: GTVnx and GTVnd (70 Gy), CTV‐1 (60 Gy), CTV‐2, and CTV‐N (56 Gy). IMRT was conducted once daily, five times per week, 33 fractions.

### 2.4 Follow up

After treatments, patients were seen for follow‐up visits once every 3 months during the first 2 years, next twice a year up to 5 years, and then once a year. The assessments were the same as pretreatment evaluations.

### 2.5 End points

The primary end points were OS (the time from treatment to death), progression‐free survival (PFS, the time from treatment to distant metastasis, locoregional recurrence, or death), DMFS (the time from treatment to distant metastasis or death), and locoregional failure‐free survival (LRFFS, the time from treatment to locoregional recurrence or death).

### 2.6 Procedures

TVRR was acquired according to the following steps. The two CT or two MRI localisations before and after IC were delivered to the radiotherapy planning system MIM Vista software (MIM corp., Version 6.8). Two experienced radiation oncologists in our department confirmed the target lesion delineation. The primary target lesions and positive cervical lymphonodus were determined in the light of the consensus.^21^ The total volumetric value of primary target lesion and positive cervical lymphonodus before and after IC were delineated as pre‐GTVsum (cm^3^) and post‐GTVsum (cm^3^), respectively. The TVRR after IC was calculated by the following formula:

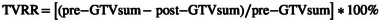




Plasma EBV DNA was detected through a quantitative polymerase chain reaction. EBV DNA levels before and after IC were defined as pre‐EBV and post‐EBV (copies/ml), respectively, which were acquired from the electronic medical record system in our hospital. The EBVCL after IC was calculated by the following formula:

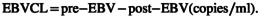




The tumor's response to IC was estimated in accordance with the RECIST 1.1.^22^ The estimation of tumor's response was conducted by using the identical CT or MRI localisations that were utilized for the TVRR calculation. The tumor's response to IC was defined as partial remission (PR), stable disease (SD), complete remission, and progressive disease.

### 2.7 Statistics

The best critical values of EBVCL and TVRR for 3‐year OS, PFS, DMFS, and LRFFS were analyzed by the receiver operating characteristic (ROC) curve. When the maximal area under ROC curve (AUC) was more than 50%, it was considered that there existed clinical significance. Spearman's method was used to analyze the correlation between the best critical value of TVRR and EBVCL. The prognostic values among EBVCL+TVRR, EBVCL, TVRR, TNM stage, and the tumor's response to IC (RECIST 1.1 criteria) were compared by ROC curve. According to the best critical values of EBVCL and TVRR (EBVCL+TVRR), participants were classified into three categories: Group A (TVRR > best critical value and EBVCL > best critical value), Group B (TVRR > best critical value and EBVCL ≤ best critical value; or TVRR ≤ best critical value and EBVCL > best critical value), and Group C (TVRR ≤ best critical value and EBVCL ≤ best critical value). And then, on the basis of the survival differences among these three groups, the participants were divided as low‐ and high‐risk groups (Figure S1). The Kaplan–Meier curves were developed for primary endpoints, the best critical values of EBVCL, TVRR, and risk groups were regarded as stratification factors. Log‐rank tests were utilized to compare the survival differences. Cox proportional hazards model was used for uni‐ and multi‐variate analysis. All hazard ratios (HRs), AUCs, and three‐year survival ratios were presented with 95% confidence intervals (CIs). All the continuous variables were presented as median (range). A two‐sided *p* value <0.05 was regarded as significant. The Medcalc (version 18.2.1) and Statistical Package for Social Sciences (SPSS) software version 23.0 (SPSS Inc.) were used to perform the statistical analyses.

## 3 RESULTS

Finally, there were 299 participants were enrolled in this study. Table 1 illustrated the patients' baseline characteristics. The clinical follow‐up ended on June 30, 2021, with a median time of 54.17 (7.33–119.2) months. For whole participants, the three‐year OS, PFS, DMFS, and LRFFS were 93.1%, 85.3%, 90.0%, and 94.1%, respectively. During clinical follow‐up, 48 (16.1%) patients suffered locoregional recurrence, 41 (13.7%) patients suffered distant metastasis, and 39 (13.0%) patients died.

**TABLE 1:** Patient characteristics

Characteristics	No. of patients (%)
Age (years)	
<48	139 (46.49)
≥48	160 (53.51)
Sex	
Male	213 (71.24)
Female	86 (28.76)
Pathological type	
Keratinizing squamous cell carcinoma	5 (1.67)
Nonkeratinizing squamous cell carcinoma	294 (98.33)
T category^a^	
1	33 (11.04)
2	76 (25.42)
3	98 (32.78)
4	92(30.76)
N category^a^	
1	21 (7.02)
2	208 (69.57)
3	70 (23.41)
TNM stage^a^	
III	179 (59.87)
IVA	120 (40.13)
IC cycles	
2	51 (17.06)
3	248 (82.94)
IC regimen	
TPF	154 (51.51)
GP	73 (24.41)
Others	72 (24.08)
Tumor response to IC according to RECIST 1.1 criteria	
PR	215 (71.91)
SD	84 (28.09)

Abbreviations: IC, induction chemotherapy; PR, partial response; RECIST, Response Evaluation Criteria in Solid Tumors. SD, stable disease.

a The 8th edition stage‐classification of UICC/AJCC.

**FIGURE 1:**
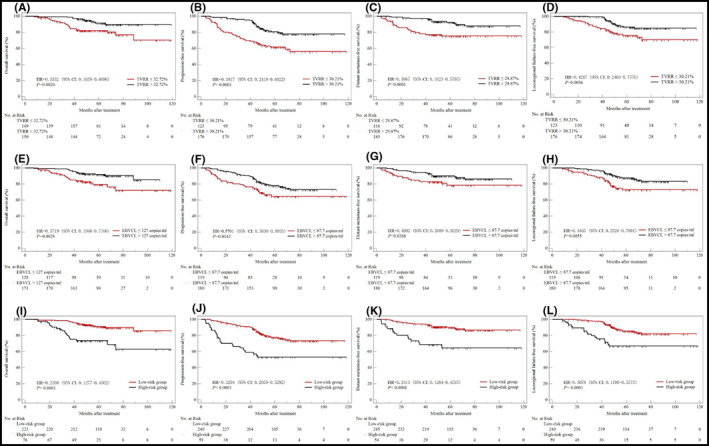
Kaplan–Meier survival curves stratified by best critical values of TVRR (A–D), EBVCL (E–H) and EBVCL+TVRR (I–L). CI, confidence interval; EBVCL, Epstein–Barr virus DNA change level; HR, hazard ratio; TVRR, tumor volume reduction ratio. *p* values were calculated with an unadjusted Cox proportional‐hazards models.

The median TVRR was 33.05% (−31.16% to 57.40%) after IC. The ROC curve analysis of TVRR on three‐year survival showed that the best critical values of TVRR was 32.72% for OS, 30.21% for PFS and LRFFS, and 29.87% for DMFS (Figure S2A through S2D). TVRR > the best critical values were significantly correlated to improved 3‐year OS (98.7% vs. 87.4% [HR = 0.3332; 95% CI: 0.1658–0.6696; *p* = 0.0020]), PFS (95.3% vs. 70.7% [HR = 0.3817; 95% CI: 0.2419–0.6022; *p* < 0.0001]), DMFS (97.2% vs. 78.1% [HR = 0.3067; 95% CI: 0.1623–0.5795; *p* = 0.0003]) and LRFFS (99.4% vs. 86.2% [HR = 0.4267; 95% CI: 0.2403–0.7576; *p* = 0.0036]) (Figure 1A–D; Table 2).

**TABLE 2:** The 3‐year survival rates in different subgroups

TVRR	> best critical value	≤ best critical value	HR (95% CI)	*p* value^a^
3‐year OS^b^	98.7%	87.4%	0.3332 (0.1658–0.6696)	0.0020
3‐year PFS^c^	95.3%	70.7%	0.3817 (0.2419–0.6022)	<0.0001
3‐year DMFS^d^	97.2%	78.1%	0.3067 (0.1623–0.5795)	0.0003
3‐year LRFFS^c^	99.4%	86.2%	0.4267 (0.2403–0.7576)	0.0036

EBVCL	> best critical value	≤ best critical value	HR (95% CI)	*p* value
3‐year OS^e^	97.7%	86.7%	0.3719 (0.1946–0.7106)	0.0028
3‐year PFS^f^	90.4%	77.5%	0.5701 (0.3638–0.8935)	0.0142
3‐year DMFS^f^	94.4%	82.9%	0.4982 (0.2689–0.9229)	0.0268
3‐year LRFFS^f^	97.2%	89.3%	0.4465 (0.2526–0.7891)	0.0055

EBVCL + TVRR	Low‐risk group	High‐risk group	HR (95% CI)	*p* value
3‐year OS	97.7%	78.3%	0.2398 (0.1277–0.4502)	<0.0001
3‐year PFS	91.1%	60.9%	0.3294 (0.2050–0.5292)	<0.0001
3‐year DMFS	94.2%	68.7%	0.2413 (0.1284–0.4535)	<0.0001
3‐year LRFFS	97.8%	77.9%	0.3078 (0.1700–0.5573)	0.0001

TNM^g^	III	IV	HR (95% CI)	*p* value
3‐year OS	95.9%	89.0%	0.5254 (0.2773–0.9956)	0.0485
3‐year PFS	88.7%	80.0%	0.5308 (0.3381–0.8335)	0.0059
3‐year DMFS	92.0%	86.9%	0.5311 (0.2864–0.9850)	0.0447
3‐year LRFFS	94.8%	92.9%	0.5272 (0.2981–0.9325)	0.0278

RECIST 1.1	PR	SD	HR (95% CI)	*p* value
3‐year OS	96.6%	84.3%	0.4555 (0.2417–0.8584)	0.0150
3‐year PFS	87.1%	80.6%	0.5760 (0.3631–0.9138)	0.0191
3‐year DMFS	91.3%	86.4%	0.5565 (0.2970–1.0428)	0.0674
3‐year LRFFS	95.7%	90.0%	0.4840 (0.2725–0.8596)	0.0133

Abbreviations: CI, confidence interval; DMFS, distant metastasis‐free survival; EBVCL, EBV DNA change level; HR, hazard ratio; LRFFS, locoregional failure‐free survival; OS, overall survivval; PFS, progression‐free survival; PR, partial response; RECIST, Response Evaluation Criteria in Solid Tumors; SD, stable disease; TVRR, tumor volume reduction ratio.

^a^ *p* values were calculated with an unadjusted Cox proportional‐hazards models.

^b^ The best critical value of TVRR was 32.72%.

^c^ The best critical value of TVRR was 30.21%.

^d^ The best critical value of TVRR was 29.87%.

^e^ The best critical value of EBVCL was 127 copies/ml.

^f^ The best critical value of EBVCL was 87.7 copies/ml.

^g^ The 8th edition stage‐classification of UICC/AJCC.

The median EBVCL was 261.5 (−4345.0 to 1380000.0) copies/ml after IC. The ROC curve analysis of EBVCL on three‐year survival showed that the best critical value of EBVCL was 127 copies/ml for OS and 87.7 copies/ml for PFS, DMFS, LRFFS (Figure S2E through S2H). EBVCL > the best critical values were significantly correlated to improved three‐year OS (97.7% vs. 86.7% [HR = 0.3719; 95% CI: 0.1946–0.7106; *p* = 0.0028]), PFS (90.4% vs. 77.5% [HR = 0.5701; 95% CI: 0.3638–0.8935; *p* = 0.0142]), DMFS (94.4% vs. 82.9% [HR = 0.4982; 95% CI: 0.2689–0.9229; *p* = 0.0268]), and LRFFS (97.2% vs. 89.3% [HR = 0.4465; 95% CI: 0.2526–0.7891; *p* = 0.0055]) (Figure 1E–H; Table 2).

The Spearman correlation coefficient between the best critical value of TVRR and EBVCL was 0.138 (*p* = 0.017), 0.140 (*p* = 0.016), 0.110 (*p* = 0.058), and 0.140 (*p* = 0.016) for OS, PFS, DMFS, and LRFFS, respectively. Although there existed positive correlation between the best critical value of EBVCL and TVRR, the correlation coefficient was pretty low (all <0.2).

**FIGURE 2:**
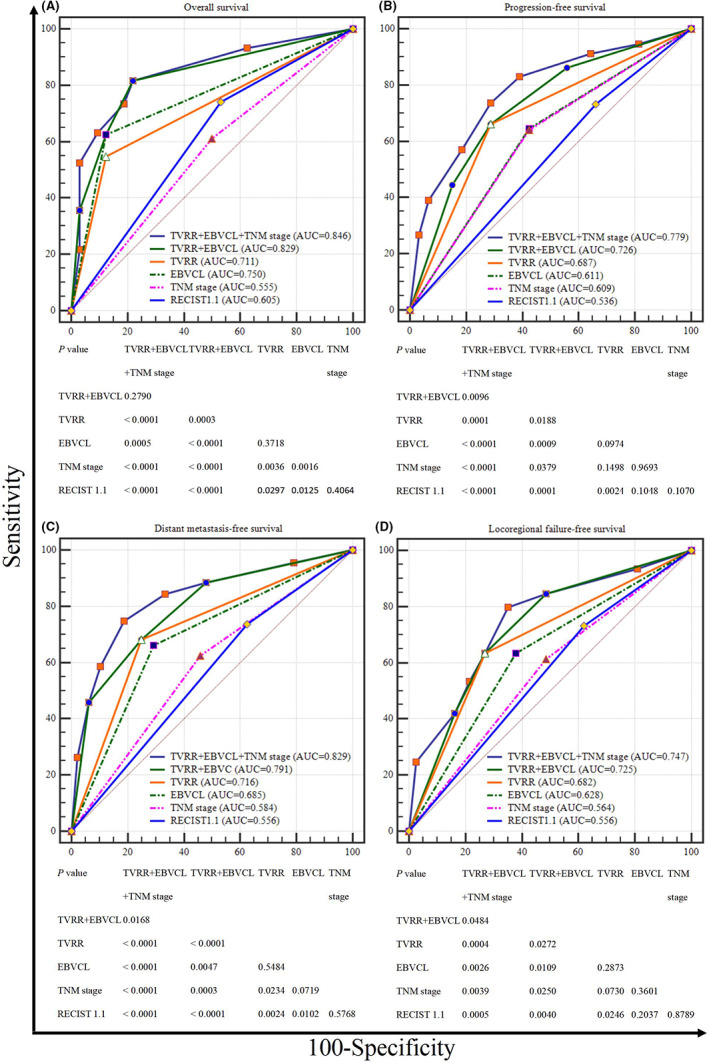
The comparision of prognostic values for EBVCL + TVRR + TNM stage, EBVCL + TVRR, EBVCL, TVRR, TNM stage, RECIST 1.1 by using receiver operating characteristic curve analysis: (A) OS, (B) PFS, (C) DMFS, (D) LRFFS. DMFS, distant metastasis‐free survival; EBVCL, Epstein–Barr virus DNA change level; LRFFS, locoregional failure‐free survival; OS, overall survivval; PFS, progression‐free survival; RECIST, Response Evaluation Criteria in Solid Tumors; TVRR, tumor volume reduction ratio.

To further verify which factor possessed the most powerful ability to predict the LANPC's prognosis, we compared the prognostic values of the following factors: EBVCL, TVRR, TNM stage, and RECIST 1.1. And found that although the prognostic value of EBVCL and TVRR were both higher than that of TNM stage and RECIST 1.1 in predicting OS, the situations were not the same for PFS, DMFS, and LRFFS. On account of the prognostic value differences were not statistically significant between EBVCL and TVRR, we decided to explore the prognostic value of the combined factor (EBVCL+TVRR). And finally, we found that EBVCL+TVRR was the most powerful predictor for three‐year OS, PFS, DMFS, and LRFFS (Figure 2). And then, we further stratified all the patients into Group A, B, C according to the best critical values of EBVCL and TVRR (EBVCL+TVRR).

Since the survival differences of three‐year OS, PFS, DMFS, and LRFFS between Groups A and B were insignificant, and there existed statistically significant survival differences between Groups A and C, or Group B and C (Table S1, Figure S3), the Groups A and B were stratified as low‐risk group, the Group C were stratified as high‐risk group (Figure S1). And the three‐year survival rates of low‐risk group were much more improved compared with high‐risk group: OS (97.7% vs. 78.3% [HR = 0.2398; 95% CI: 0.1277–0.4502; *p* < 0.0001]), PFS (91.1% vs. 60.9% [HR = 0.3294; 95% CI: 0.2050–0.5292; *p* < 0.0001]), DMFS (94.2% vs. 68.7% [HR = 0.2413; 95% CI: 0.1284–0.4535; *p* < 0.0001]), and LRFFS (97.8% vs. 77.9% [HR = 0.3078; 95% CI: 0.1700–0.5573; *p* = 0.0001]) (Figure 1I– L; Table 2). Subgroup analyses of TNM Stages III and IV revealed that low‐risk population possessed superior 3‐year OS and PFS compared with high‐risk population in both subgroups (Table S2).

Besides, the patients' survival comparision stratified by TNM stage (III vs. IVA) and tumor's response to IC according to RECIST 1.1 criteria (PR vs. SD) were also performed, the 3‐year survival differences of III versus IVA (Figure S4) or PR versus SD (Figure S5) were all statistically significant, excepting for the DMFS stratified by RECIST 1.1 criteria (PR vs. SD) (Table 2).

Univariate analyses revealed EVBCL, TVRR, EBVCL+TVRR, T category, N category, TNM stage, and RECIST1.1 were all prognostic indicators for OS; EVBCL, TVRR, EBVCL+TVRR, T category, TNM stage, and RECIST1.1 were all prognostic indicators for PFS; EVBCL, TVRR, EBVCL+TVRR and TNM stage were all prognostic factors for DMFS; EVBCL, TVRR, EBVCL+TVRR, T category, TNM stage, and RECIST1.1 were all prognostic factors for LRFFS (Table S3). Although radiotherapy was interrupted in 19 patients, including seven cases' interruption time ≤ 3 days, 11 cases' interruption time between 4 and 7 days, and one case’ interruption time >7 days, there existed no significant difference in survival time between patients with and without radiotherapy interruption (Table S3). In multivariate analyses, after adjusted by TNM stage, RECIST 1.1 criteria and cycles of IC, EBVCL + TVRR was testified as an independent indicator of the prognosis for OS, PFS, DMFS, and LRFFS (Table 3).

**TABLE 3:** Multivariate analyses

Variable	HR (95% CI)	*p* value^a^
Overall survival		
EBVCL+TVRR (Low‐risk group vs. High‐risk group)	0.2250 (0.1169–0.4331)	<0.0001
TNM stage (III vs. IV)	0.5380 (0.2799–1.0343)	0.0631
RECIST 1.1 (PR vs. SD)	0.5277 (0.2776–1.0033)	0.0512
IC cycles (3 vs. 2)	0.5813 (0.2461–1.3729)	0.2160
Progression‐free survival		
EBVCL+TVRR (Low‐risk group vs. High‐risk group)	0.3130 (0.1932–0.5073)	<0.0001
TNM stage (III vs. IV)	0.5553 (0.3515–0.8772)	0.0117
RECIST 1.1 (PR vs. SD)	0.5963 (0.3746–0.9489)	0.0292
IC cycles (3 vs. 2)	0.6951 (0.3836–1.2595)	0.2304
Distant metastasis‐free survival		
EBVCL+TVRR (Low‐risk group vs. High‐risk group)	0.2402 (0.1272–0.4536)	<0.0001
TNM stage (III vs. IV)	0.5563 (0.2966–1.0436)	0.0677
RECIST 1.1 (PR vs. SD)	0.6087 (0.3231–1.1469)	0.1245
IC cycles (3 vs. 2)	0.9076 (0.3750–2.1967)	0.8297
Locoregional failure‐free survival		
EBVCL+TVRR (Low‐risk group vs. High‐risk group)	0.2861 (0.1561–0.5245)	<0.0001
TNM stage (III vs. IV)	0.5733 (0.3216–1.0222)	0.0593
RECIST 1.1 (PR vs. SD)	0.4916 (0.2752–0.8783)	0.0165
IC cycles (3 vs. 2)	0.6746 (0.3206–1.4195)	0.2997

Abbreviations: CI, confidence interval; EBVCL, EBV DNA change level; HR, hazard ratio; IC, induction chemotherapy; PR, partial response; RECIST, Response Evaluation Criteria in Solid Tumors; SD, stable disease; TVRR, tumor volume reduction ratio.

^a^ *p* values were calculated with an adjusted Cox proportional‐hazards models.

To identify whether EBVCL + TVRR could boost the predictive value of TNM stage, we also explored the prognostic value of EBVCL + TVRR + TNM stage, and found that the AUC values of EBVCL + TVRR + TNM stage were all significantly larger than that of TNM stage alone for OS, PFS, DMFS and LRFFS (Figure).

## 4 DISCUSSION

In the present study, we explored the prognosis predicting ability of EBV DNA change level combined with tumor volume reduction ratio after inductive chemotherapy in LANPC for the first time, and finally developed a new prognostic predictor (EVBCL+TVRR), which stratified LANPC patients into low‐ and high‐risk group and could better indicate the patient's prognosis compared with other factors commonly used in the clinical practice, such as TNM stage system or RECIST 1.1 criteria.

Up to present, these factors including TNM stage system, RECIST 1.1 criteria, tumor load, and EVB DNA are all prognostic predictors for LANPC, but the most powerful indicator has not been revealed. And in our previous study, we have verified the prognostic value of TVRR for LANPC.^19^ Besides, EBV DNA level is also a meaningful and commonly used indicator for LANPC in the clinical work, but most of the studies only investigated the prognostic value of the static EBV DNA level, and ignored the value of dynamic change of EBV DNA during the treatment for LANPC. Based on the above current situations, we decided to find out the most valuable prognosis predicting factor for LANPC among TNM stage system, RECIST 1.1 criteria, TVRR, and EBVCL after IC. And we found that the prognostic value of EBVCL and TVRR were both higher than that of TNM stage and RECIST 1.1 in predicting OS, but the situations were not the same for PFS, DMFS, and LRFFS. Considering there existed no statistical significant prognosis predicting difference between EBVCL and TVRR for three‐year survival (all *p* > 0.05), we further explored the prognostic value of the combined factor (EBVCL+TVRR), and finally revealed the combined factor possessed the most powerful prognosis predicting ability for OS, PFS, DMFS, and LRFFS when compared with EBVCL, TVRR, TNM stage or RECIST 1.1 (Figure 2). Contrasted with our previously published results, there mainly exists the following similarities and differences.^19^ Firstly, although the number of included patient of our current study (299) was much more than that of our previous study (70), and the survival time used for ROC curve analyses was 3 and 2 years for the present and previous study, respectively, the mean best critical values of TVRR for PFS, LRFFS and DMFS in our current (30.10%) and previous (26.08%) study were both approximately equal to 30%, which also illustrated the evaluation model's stability of our studies. Secondly, what should be noticed was that the mean AUC value of TVRR for PFS, LRFFS, and DMFS in this present study was larger than that of the previous study (0.723 vs. 0.647), as AUC value represents the clinical significance of the best critical value, we speculated that based on the enlarged sample size of our present study, the results we concluded became more powerful and credible. Thirdly, on the basis of our previous study, we not only explored the prognostic value of TVRR after IC, but also verified the prognostic value of EBVCL after IC for LANPC for the first time, furthermore, after combining the two factors together, the newly developed indicator (EBVCL+TVRR) possessed the most powerful prognosis predicting ability when compared with EBVCL or TVRR alone, TNM stage system and RECIST 1.1 criteria. Besides, we further identified that the addition of EBVCL + TVRR to TNM stage could significantly boost the ability to predict the prognosis of LANPC.

Recently, a similar study conducted by Huiyun Yang et al. also explored the prognostic value of TVRR after inductive chemotherapy in LANPC.^23^ The best critical value of TVRR in their study was 12.6%, which was smaller than the results in ours (32.72% for OS, 30.21% for PFS and LRFFS, 29.87% for DMFS), and we speculated that this diversity was mainly caused by the different IC regimens and cycles. On the one hand, although there both existed TPF, TP, and PF regimens in their and our study, we also included patients who received GP, which accounted for 24.41% (73/299) of the whole patients, as we all know, there are many kinds of IC regimens for LANPC at present, but the optimal regimen still remains unknown, and the lesion's response to different regimen may vary a lot; on the other hand, we excluded the patients who only underwent 1 cycle of IC, which was also enrolled in their study, so the heterogeneity among different IC regimens and cycles might lead to the discrepant best critical value of TVRR after IC. Furthermore, the analyses methods of the best critical value for different survival endpoints between their and our study were not the same, although both the two study used ROC analysis, they used the best critical value of TVRR for DFS (disease‐free survival) as the stratification factor for OS, LRFFS, and DMFS as well, however, we calculated the best critical value of TVRR for OS, PFS, LRFFS, and DMFS separately. It should be also noted that when combined TVRR and TNM stage together in their study, excepting for DFS and LRFFS, the prognosis predicting ability of OS was not improved when compared with TNM alone; but in our study, when TVRR was united with EBVCL, the prognosis predicting power was significantly ameliorated for all survival endpoints (OS, PFS, LRFFS, and DMFS) when compared with either TNM stage or RECIST 1.1.

But we must admit that the different best critical values of EBVCL and TVRR for different survival endpoints might bring some inconvenience for the use of our results in the clinic, but considering the accuracy of the results, we decided to use the exact best critical values for OS, PFS, DMFS, and LRFFS calculated by ROC curve analyses. In our study, the best critical values of TVRR were all approximately equal to 30% (32.72% for OS, 30.21% for PFS and LRFFS, and 29.87% for DMFS). But the best critical value of EBVCL for OS was larger than that of PFS, DMFS, and LRFFS (127 copies/ml for OS, 87.7 copies/ml for PFS, DMFS, and LRFFS), which to certain extent revealed that only a relatively larger EBV‐DNA decrease after IC could guarantee a long overall survival time.

There also exists some other studies who developed predictive models of prognosis for LANPC patients who received IC + CCRT. A recent research conducted by Fo‐Ping Chen et al. developed a circulating EBV DNA level based prognostication predicting system for LANPC.^24^ In their study, the EBV DNA levels before and after IC and the N category were combined, the participants were stratified as high‐, low‐ and median‐risk group, the 5‐year OS ratio was 88.1%, 79.2% and 66.9%, respectively. In the ROC analyses, our AUC values of TNM stage for OS and DMFS were 0.555 and 0.584, respectively, which were similar with theirs (0.562 for OS and DMFS). And compared with the AUC values (0.631, 0.659 for OS and DMFS, respectively) of their combined prognosis predicting system (EBV DNA level + N category), our combined factor (EBVCL + TVRR) seems to possess a superior prognostic ability (AUC: 0.829, 0.791 for OS and DMFS, respectively). We speculate the reason is that the dynamic changes of tumor load and EBV DNA level after IC might better mirror the primary lesion's response to treatment than the static status of tumor and EBV DNA level. Another study performed by Li‐Ting Liu et al. also divided NPC patients with III‐IVb stages into low‐, median‐ and high‐risk group based on the EBV DNA level and N category, and explored which group would benefit from CCRT, IC + CCRT or CCRT+adjuvant chemotherapy (ACT), and they found that, in low‐risk group, patients who received IC + CCRT possessed less distant‐metastasis risk compared with who only received CCRT alone, the survival differences in the median‐ and high‐risk groups did not show any statistical significance.^25^ But in our present study, the three‐year overall survival difference between low‐ and high‐risk group was statistically significant.

Besides, Qiu‐Yan Chen et al. combined the pretreatment EBV DNA with tumor volume to predict the early stage NPC patient's prognosis in the IMRT era, they divided patients into low‐ and high‐risk groups based on the tumor volume and EBV DNA level, and found that low‐risk group possessed higher three‐year PFS, LRFFS, and DMFS ratios compared with high‐risk group.^26^ Notably, these studies^25^, ^26^ all paid attention to the static status of tumor load and EBV DNA level before treatment, our current study focused on the dynamic changes of tumor load (TVRR) and EBV DNA (EBVCL) after IC, and developed a better prognostic indicator for LANPC patients on the basis of the combined factor (EBVCL+TVRR) for the first time, which may provide some reference for the LANPC's treatment after IC in the clinical practice.

As the tumor could shrink in different degrees after IC, and it has been validated that the addition of IC to CCRT could improve the survival of LANPC patients.^4^, ^5^ Some studies explored the feasibility of de‐intensification strategy after IC. Hongru Yang et al found the quality of life of LANPC patients treated with reduced target volumes/radiation doses IMRT after IC was improved with decreased doses received by normal tissues and similar 3‐year OS, PFS, LRFFS and DMFS rates, compared with who treated with none reduced target volumes/radiation doses IMRT after IC.^27^ But the international guideline for the delineation of clinical target volume for NPC still recommends the tumor volume before IC should receive the full therapeutic dose regardless of shrinkage after IC.^28^ And there exists ongoing clinical trial to further identify the feasibility of reduced target volumes/radiation doses IMRT (NCT03668730). In terms of this de‐intensification strategy after IC for LANPC, our study might provide some guidance for the following treatment after IC of LANPC. For low‐risk patients, the reduced target volumes/radiation doses IMRT might be appropriate in accordance with the post‐IC tumor volume; for high‐risk patients, the delineation of target volumes would be better on the basis of pre‐IC tumor volume. But there still needs further study to testify the reference value of our results in terms of this de‐intensification strategy in the future.

Our study exists some limitations. First, there may be selective bias due to the retrospective nature of this research, and we strictly included eligible patients according to the inclusion criteria to minimize this bias. Secondly, objectively speaking, obtaining TVRR is relatively complex according to the present technical level, which may bring obstacles to the wide use of our combined prognostic predictor, but as the artificial intelligence (AI) has been being rapidly improved, the automatic target lesion's delineation maybe come true someday in the future, which might make the acquirement of TVRR more easier. Li Lin et al constructed an AI contouring tool to automatically delineate the primary gross tumor volume of NPC, which could effectively improve the target delineation accuracy with a reduced contouring time by 39.4%.^29^ The SC‐DenseNet model developed by Liangru Ke et al showed promising ability in the automatic delineation of tumor area in NPC.^30^ Another novel semi‐supervised learning framework for target volume delineation in NPC constructed by Wenjun Liao et al exhibited high delineation accuracy with a 60% improved delineating efficiency.^31^ Finally, most of the radiation oncologists in our department usually only arranged once localisation after IC, the proportion of patients who received twice localisations before and after IC was small, which indeed limited the sample size of our study, so more larger prospective researches are necessary to testify the repeatability and generality of the combined predictor (EVBCL + TVRR) for LANPC in the future.

In conclusion, the newly developed indicator (EBVCL + TVRR), with a great potential clinical applications under the development of artificial intelligence, possessed more powerful prognosis predicting ability than TNM stage or RECIST 1.1 criteria, which could better predict the LANPC patient's survival after inductive chemotherapy, thus providing some guidance for the following treatment in clinical practice to achieve a more personalized precision medicine. For instance, for high‐risk population, a more aggressive therapeutic regimen such as complementary radiation dose for primary tumor or addition of adjuvant chemotherapy after CCRT might be considered, which may further prolong the survival time on the basis of IC followed by CCRT; and for low‐risk population, the reduced target volumes/radiation doses IMRT based on the post‐IC tumor volume might be appropriate. And in the future, we will further explore the specific guidance ability of the new indicator in the formulation of the subsequent treatment strategies after IC for LANPC patients.

## AUTHOR CONTRIBUTIONS

Zhong‐zheng Xiang, and Lei Liu had complete access to the whole data in this research and were responsible for the integrality and correctness of the data and analyses. Drs Zhong‐zheng Xiang, Tao He and Yuan‐yuan Zeng contributed equally to this study. Concept and design: Lei Liu, Zhong‐zheng Xiang, Tao He. Drafting of the manuscript: Zhong‐zheng Xiang, Tao He. Critical revision of the manuscript for important intellectual content: Lei Liu, Fang Liu, Bian‐fei Shao. Statistical analysis: Tao He, Zhong‐zheng Xiang, Tian Yang, Jia‐chun Ma, Xi‐ran Wang, Si‐ting Yu. Supervision: Lei Liu.

## FUNDING INFORMATION

None.

## CONFLICT OF INTEREST

No conflict of interest was declared by the authors.

## Supporting information


Figure S1

Figure S2

Figure S3

Figure S4

Figure S5

Table S1

Table S2

Table S3

## Data Availability

Data sharing is not applicable to this article as no new data were created or analyzed in this study.
