# Supplementary material for: Epstein–Barr virus DNA change level combined with tumor volume reduction ratio after inductive chemotherapy as a better prognostic predictor in locally advanced nasopharyngeal carcinoma
Source: Cancer Med. 2022 Jul 19;12(2):1102–13. doi: 10.1002/cam4.4964 (PMC9883421; doi:10.1002/cam4.4964)
Supplement: Supplementary file 1 — Figure S1 [file CAM4-12-1102-s003.pdf]

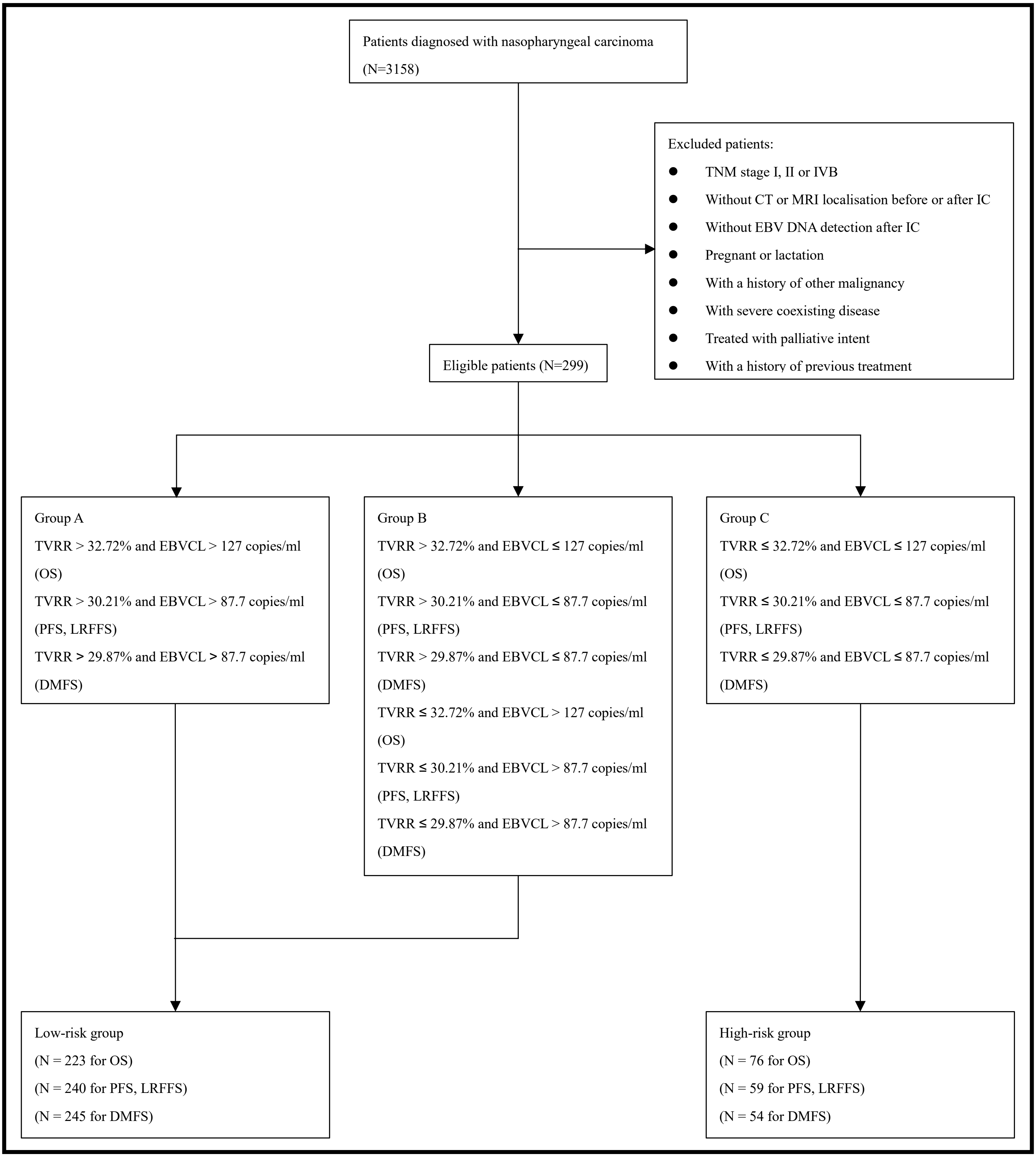
Patients diagnosed with nasopharyngeal carcinoma (N=3158)
Excluded patients:
TNM stage I, II or IVB
Without CT or MRI localisation before or after IC
Without EBV DNA detection after IC
Pregnant or lactation
With a history of other malignancy
With severe coexisting disease
Treated with palliative intent
With a history of previous treatment
Eligible patients (N=299)
Group A
TVRR > 32.72% and EBVCL > 127 copies/ml (OS)
TVRR > 30.21% and EBVCL > 87.7 copies/ml (PFS, LRFFS)
TVRR > 29.87% and EBVCL > 87.7 copies/ml (DMFS)
Group B
TVRR > 32.72% and EBVCL ≤ 127 copies/ml (OS)
TVRR > 30.21% and EBVCL ≤ 87.7 copies/ml (PFS, LRFFS)
TVRR > 29.87% and EBVCL ≤ 87.7 copies/ml (DMFS)
TVRR ≤ 32.72% and EBVCL > 127 copies/ml (OS)
TVRR ≤ 30.21% and EBVCL > 87.7 copies/ml (PFS, LRFFS)
TVRR ≤ 29.87% and EBVCL > 87.7 copies/ml (DMFS)
Group C
TVRR ≤ 32.72% and EBVCL ≤ 127 copies/ml (OS)
TVRR ≤ 30.21% and EBVCL ≤ 87.7 copies/ml (PFS, LRFFS)
TVRR ≤ 29.87% and EBVCL ≤ 87.7 copies/ml (DMFS)
Low-risk group
(N = 223 for OS)
(N = 240 for PFS, LRFFS)
(N = 245 for DMFS)
High-risk group
(N = 76 for OS)
(N = 59 for PFS, LRFFS)
(N = 54 for DMFS)
