# Supplementary figures and images for: Epstein–Barr virus DNA change level combined with tumor volume reduction ratio after inductive chemotherapy as a better prognostic predictor in locally advanced nasopharyngeal carcinoma
Source: Cancer Med. 2022 Jul 19;12(2):1102–13. doi: 10.1002/cam4.4964 (PMC9883421; doi:10.1002/cam4.4964)

Sensitivity

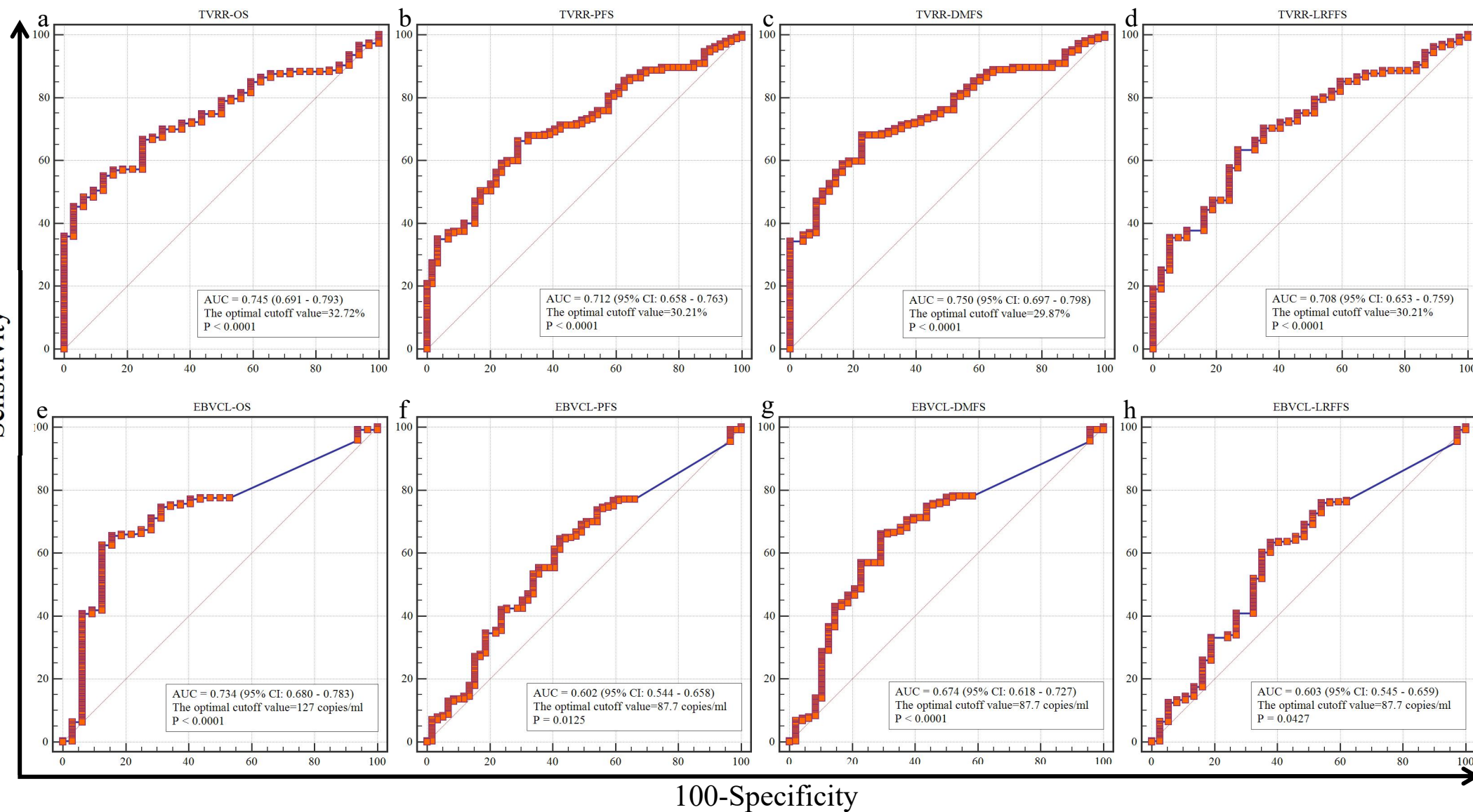

100-Specificity

Supplement: Supplementary file 2 — Figure S2 [file CAM4-12-1102-s006.pdf]

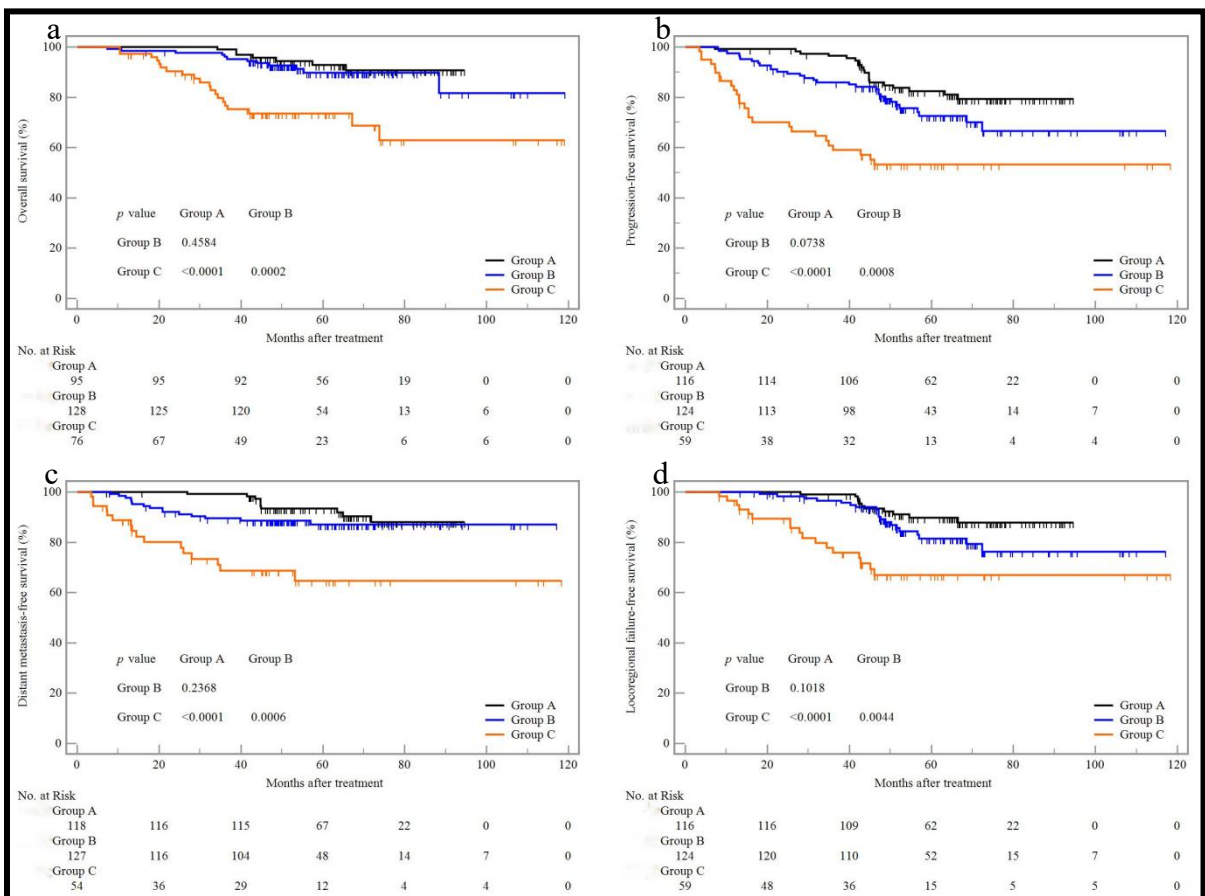

Supplement: Supplementary file 3 — Figure S3 [file CAM4-12-1102-s001.pdf]

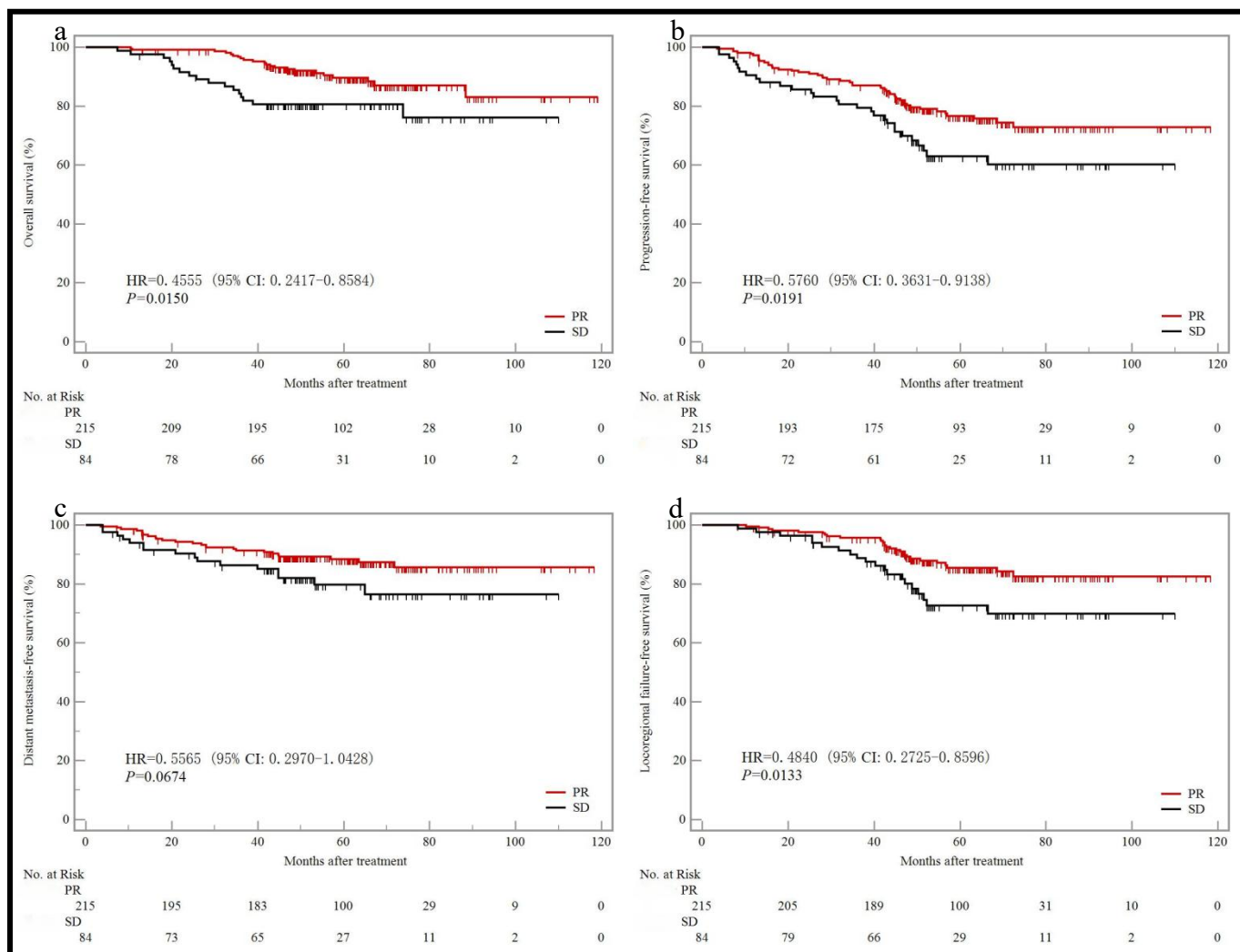

Supplement: Supplementary file 5 — Figure S5 [file CAM4-12-1102-s002.pdf]
