# Supplementary material for: Epstein–Barr virus DNA change level combined with tumor volume reduction ratio after inductive chemotherapy as a better prognostic predictor in locally advanced nasopharyngeal carcinoma
Source: Cancer Med. 2022 Jul 19;12(2):1102–13. doi: 10.1002/cam4.4964 (PMC9883421; doi:10.1002/cam4.4964)
Supplement: Supplementary file 4 — Figure S4 [file CAM4-12-1102-s007.pdf]

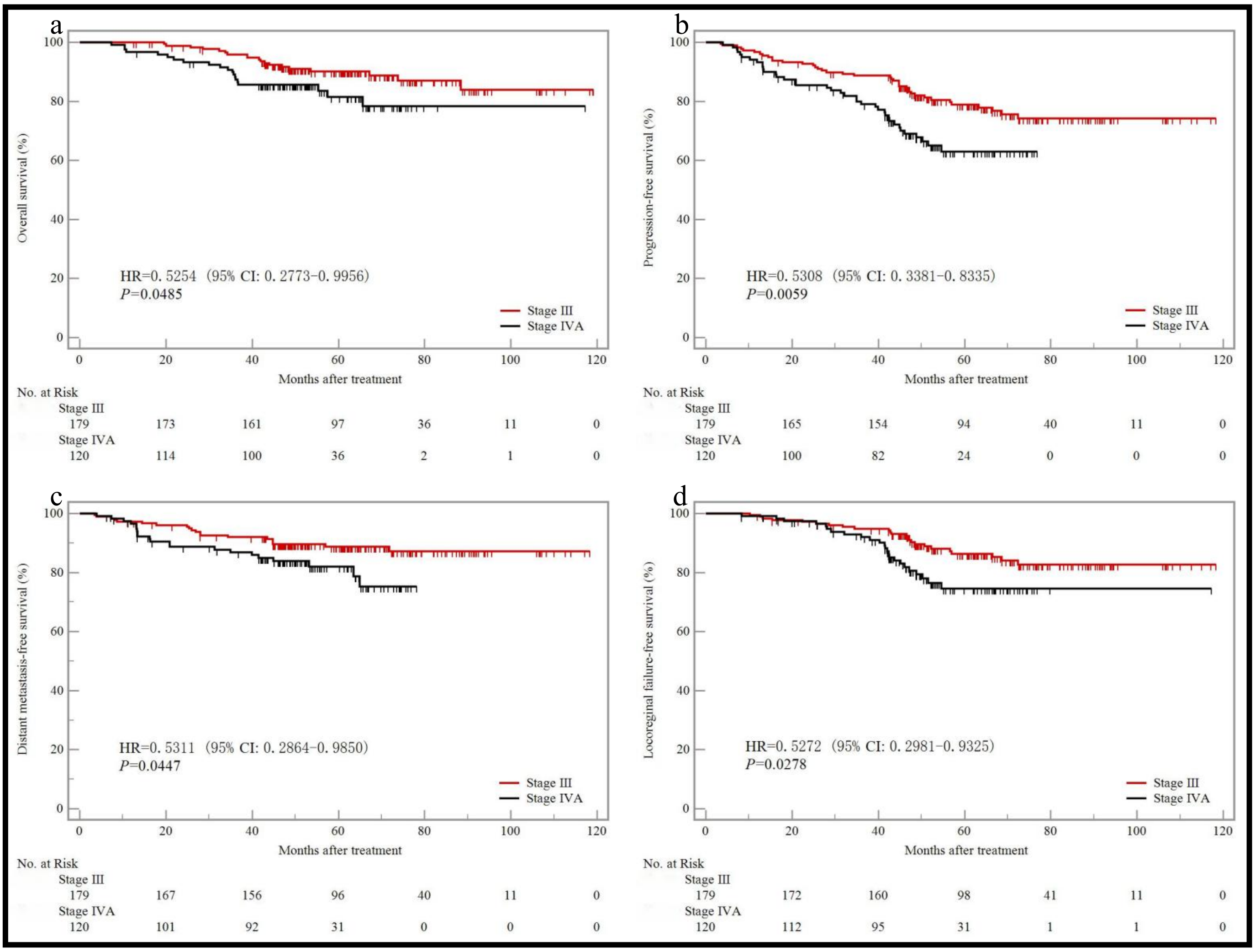
a
Overall survival (%)
HR=0. 5254 (95% CI: 0. 2773-0. 9956)
P=0.0485
Stage III
Stage IVA
Months after treatment
No. at Risk
Stage III
179 173 161 97 36 11 0
Stage IVA
120 114 100 36 2 1 0
b
Progression-free survival (%)
HR=0. 5308 (95% CI: 0. 3381-0. 8335)
P=0.0059
Stage III
Stage IVA
Months after treatment
No. at Risk
Stage III
179 165 154 94 40 11 0
Stage IVA
120 100 82 24 0 0 0
c
Distant metastasis-free survival (%)
HR=0. 5311 (95% CI: 0. 2864-0. 9850)
P=0.0447
Stage III
Stage IVA
Months after treatment
No. at Risk
Stage III
179 167 156 96 40 11 0
Stage IVA
120 101 92 31 0 0 0
d
Locoreginal failure-free survival (%)
HR=0. 5272 (95% CI: 0. 2981-0. 9325)
P=0.0278
Stage III
Stage IVA
Months after treatment
No. at Risk
Stage III
179 172 160 98 41 11 0
Stage IVA
120 112 95 31 1 1 0
