# Supplementary material for: Epstein–Barr virus DNA change level combined with tumor volume reduction ratio after inductive chemotherapy as a better prognostic predictor in locally advanced nasopharyngeal carcinoma
Source: Cancer Med. 2022 Jul 19;12(2):1102–13. doi: 10.1002/cam4.4964 (PMC9883421; doi:10.1002/cam4.4964)
Supplement: Supplementary file 6 — Table S1 [file CAM4-12-1102-s005.doc]

Supplementary TABLE 1 The 3-year survival differences among group A, B and C

| **Variable** | **3-year survival** | **HR (95% CI)** | ***P* value^*^** |
| --- | --- | --- | --- |
| **Group A vs. B** |  |  |  |
| OS | 98.9% vs. 96.8% | 0.7131 (0.3518-1.4456) | 0.4584 |
| PFS | 96.5% vs. 86.0% | 0.6201 (0.3825-1.0053) | 0.0738 |
| DMFS | 99.1% vs. 89.6% | 0.6378 (0.3302-1.2321) | 0.2368 |
| LRFFS | 99.1% vs. 96.6% | 0.5573 (0.3022-1.0277) | 0.1018 |
| **Group A vs. C** |  |  |  |
| OS | 98.9% vs. 78.3% | 0.1980 (0.0828-0.4735) | <0.0001 |
| PFS | 96.5% vs. 60.9% | 0.2551 (0.1296-0.5025) | <0.0001 |
| DMFS | 99.1% vs. 68.7% | 0.1910 (0.0738-0.4947) | <0.0001 |
| LRFFS | 99.1% vs. 77.9% | 0.2213 (0.0937-0.5231) | <0.0001 |
| **Group B vs. C** |  |  |  |
| OS | 96.8% vs. 78.3% | 0.2776 (0.1195-0.6449) | 0.0002 |
| PFS | 86.0% vs. 60.9% | 0.4114 (0.2082-0.8130) | 0.0008 |
| DMFS | 89.6% vs. 68.7% | 0.2995 (0.1151-0.7796) | 0.0006 |
| LRFFS | 96.6% vs. 77.9% | 0.3972 (0.1683-0.9372) | 0.0044 |

Abbreviations: HR, hazard ratio; CI, confidence interval; OS, overall survivval; PFS, progression-free survival; DMFS, distant metastasis-free survival; LRFFS, locoregional failure-free survival.

Group A: TVRR ( tumor volume reduction ratio) > best critical value and EBVCL (EBV DNA change level) > best critical value.

Group B: TVRR > best critical value and EBVCL ≤ best critical value; or TVRR ≤ best critical value and EBVCL > best critical value.

Group C: TVRR ≤ best critical value and EBVCL ≤ best critical value.

^*^*P* values were calculated by log-rank tests.
