# Supplementary material for: Epstein–Barr virus DNA change level combined with tumor volume reduction ratio after inductive chemotherapy as a better prognostic predictor in locally advanced nasopharyngeal carcinoma
Source: Cancer Med. 2022 Jul 19;12(2):1102–13. doi: 10.1002/cam4.4964 (PMC9883421; doi:10.1002/cam4.4964)
Supplement: Supplementary file 7 — Table S2 [file CAM4-12-1102-s004.docx]

| **Factors** | **3-year Survival rate of low-risk group (%), (no. of patients)** | **3-year Survival rate of high-risk group (%), (no. of patients)** | **HR (95% CI)** | ***P* value*** |
| --- | --- | --- | --- | --- |
| **Overall survival** |  |  |  |  |
| TNM stage III | 99.2 (*n* = 134) | 84.9 (*n* = 45) | 0.2717 (0.0876-0.8425) | 0.0023 |
| TNM stage IV | 95.5 (*n* = 89) | 72.6 (*n* = 31) | 0.2101 (0.0708-0.6229) | 0.0001 |
| **Progression-free survival** |  |  |  |  |
| TNM stage III | 95.8 (*n* = 144) | 59.2 (*n* = 35) | 0.2513 (0.1005-0.6283) | < 0.0001 |
| TNM stage IV | 83.8 (*n* = 96) | 64.2 (*n* = 24) | 0.4658 (0.1955-0.9940) | 0.0284 |
| **Distant metastasis-free survival** |  |  |  |  |
| TNM stage III | 96.5 (*n* = 146) | 69.9 (*n* = 43) | 0.1940 (0.0542-0.6940) | < 0.0001 |
| TNM stage IV | 90.6 (*n* = 99) | 67.1 (*n* = 21) | 0.3108 (0.0887-1.0887) | 0.0074 |
| **Locoregional failure-free survival** |  |  |  |  |
| TNM stage III | 99.3 (*n* = 144) | 75.3 (*n* = 35) | 0.2202 (0.0685-0.7077) | 0.0001 |
| TNM stage IV | 95.6 (*n* = 96) | 81.7 (*n* = 24) | 0.4684 (0.1580-1.3886) | 0.0835 |

**Supplementary TABLE 2** The 3-year survival rates in TNM stage III and IV subgroups

Abbreviations: HR, hazard ratio; CI, confidence interval

The TNM stage was performed according to the 8th edition stage-classification of UICC/AJCC

*P values were calculated with an unadjusted Cox proportional-hazards models.
