# Supplementary material for: Epstein–Barr virus DNA change level combined with tumor volume reduction ratio after inductive chemotherapy as a better prognostic predictor in locally advanced nasopharyngeal carcinoma
Source: Cancer Med. 2022 Jul 19;12(2):1102–13. doi: 10.1002/cam4.4964 (PMC9883421; doi:10.1002/cam4.4964)
Supplement: Supplementary file 8 — Table S3 [file CAM4-12-1102-s008.doc]

Supplementary TABLE 3 Univariate analyses

| **Variable** | **HR (95% CI)** | ***P* value**^*^ |
| --- | --- | --- |
| **Overall survival** |  |  |
| age (<48 years vs. ≥48 years) | 0.9697 (0.5165-1.8205) | 0.9238 |
| Sex (female vs. male) | 0.9238 (0.4796-1.9383) | 0.9184 |
| T category (T1/2 vs. T3/4) | 0.3865 (0.1705-0.8763) | 0.0228 |
| N category (N1/2 vs. N3) | 0.4753 (0.2493-0.9063) | 0.0239 |
| TNM stage (III vs. IVA) ^a^ | 0.5254 (0.2773-0.9956) | 0.0485 |
| RECIST1.1 (PR vs. SD) | 0.4555 (0.2417-0.8584) | 0.0150 |
| IC cycles (3 vs. 2) | 0.9308 (0.4105-2.1104) | 0.8636 |
| With vs. without radiotherapy interruption | 0.8248 (0.2294-2.9647) | 0.7480 |
| TVRR (> 32.72% vs. ≤32.72%) | 0.3332 (0.1658-0.6696) | 0.0020 |
| EBVCL ( >127 copies/ml vs. ≤127 copies/ml) | 0.3719 (0.1946-0.7106) | 0.0028 |
| EBVCL+TVRR (Low- vs. High-risk group) | 0.2398 (0.1277-0.4502) | < 0.0001 |
| **Progression-free survival** |  |  |
| age (<48 years vs. ≥48 years) | 0.8631 (0.5495-1.3557) | 0.5228 |
| Sex (female vs. male) | 0.8734 (0.5392-1.4148) | 0.5824 |
| T category (T1/2 vs. T3/4) | 0.4882 (0.2849-0.8368) | 0.0091 |
| N category (N1/2 vs. N3) | 0.6532 (0.4009-1.0643) | 0.0873 |
| TNM stage (III vs. IVA)^a^ | 0.5308 (0.3381-0.8335) | 0.0059 |
| RECIST1.1 (PR vs. SD) | 0.5760 (0.3631-0.9138) | 0.0191 |
| IC cycles (3 vs. 2) | 0.9159 (0.5131-1.6350) | 0.7663 |
| With vs. without radiotherapy interruption | 0.7363 (0.2844-1.9061) | 0.4695 |
| TVRR (> 30.21% vs. ≤30.21%) | 0.3817 (0.2419-0.6022) | < 0.0001 |
| EBVCL (>87.7 copies/ml vs. ≤87.7 copies/ml) | 0.5701 (0.3638-0.8935) | 0.0142 |
| EBVCL+TVRR (Low- vs. High-risk group) | 0.3294 (0.2050-0.5292) | < 0.0001 |
| **Distant metastasis-free survival** |  |  |
| age (<48 years vs. ≥48 years) | 0.9718 (0.5259-1.7957) | 0.9273 |
| Sex (female vs. male) | 0.7127 (0.3402-1.4932) | 0.3695 |
| T category (T1/2 vs. T3/4) | 0.4964 (0.2369-1.0403) | 0.0635 |
| N category (N1/2 vs. N3) | 0.6861 (0.3500-1.3446) | 0.2724 |
| TNM stage (III vs. IVA)^a^ | 0.5311 (0.2864-0.9850) | 0.0447 |
| RECIST1.1 (PR vs. SD) | 0.5565 (0.2970-1.0428) | 0.0674 |
| IC cycles (3 vs. 2) | 0.8512 (0.3580-2.0239) | 0.7154 |
| With vs. without radiotherapy interruption | 0.5835 (0.1597-2.1317) | 0.2998 |
| TVRR (>29.87% vs. ≤29.87%) | 0.3067 (0.1623-0.5795) | 0.0003 |
| EBVCL (>87.7 copies/ml vs. ≤87.7 copies/ml) | 0.4982 (0.2689-0.9229) | 0.0268 |
| EBVCL+TVRR (Low- vs. High-risk group) | 0.2413 (0.1284-0.4535) | < 0.0001 |
| **Locoregional failure-free survival** |  |  |
| age (<48 years vs. ≥48 years) | 0.8372 (0.4715-1.4863) | 0.5439 |
| Sex (female vs. male) | 0.6071 (0.3403-1.083) | 0.0910 |
| T category (T1/2 vs. T3/4) | 0.4707 (0.2344-0.9451) | 0.0341 |
| N category (N1/2 vs. N3) | 0.6821 (0.3661-1.2721) | 0.2292 |
| TNM stage (III vs. IVA)^a^ | 0.5272 (0.2981-0.9325) | 0.0278 |
| RECIST1.1 (PR vs. SD) | 0.4840 (0.2725-0.8596) | 0.0133 |
| IC cycles (3 vs. 2) | 0.8880 (0.4300-1.8338) | 0.7481 |
| With vs. without radiotherapy interruption | 0.9542 (0.3036-2.9988) | 0.9373 |
| TVRR (> 30.21% vs. ≤30.21%) | 0.4267 (0.2403-0.7576) | 0.0036 |
| EBVCL (>87.7 copies/ml vs. ≤87.7 copies/ml) | 0.4465 (0.2526-0.7891) | 0.0055 |
| EBVCL+TVRR (Low- vs. High-risk group) | 0.3078 (0.1700-0.5573) | 0.0001 |

Abbreviations: HR, hazard ratio; CI, confidence interval; RECIST, Response Evaluation Criteria in Solid Tumors; PR, partial response; SD, stable disease; IC, induction chemotherapy; TVRR, tumor volume reduction ratio; EBVCL, EBV DNA change level.

^*^ *P* values were calculated with an unadjusted Cox proportional-hazards models.

^a^ The 8th edition stage-classification of UICC/AJCC.
